# Study on the Mechanism of RuHaoDaShi Granules in Treating H1N1 Viral Pneumonia Based on Network Pharmacology and Experimental Validation

**DOI:** 10.3390/pathogens14080834

**Published:** 2025-08-21

**Authors:** Aixin Chen, Tianhang Chen, Yu He, Jiehong Yang, Haitong Wan

**Affiliations:** 1 School of Life Science, Zhejiang Chinese Medical University, Hangzhou 310053, China; cax17373568112@163.com; 2 School of Basic Medical Sciences, Zhejiang Chinese Medical University, Hangzhou 310053, China; chentianhang@zcmu.edu.cn (T.C.); yjhong@zcmu.edu.cn (J.Y.); 3 School of Pharmaceutical Sciences, Zhejiang Chinese Medical University, Hangzhou 310053, China; heyu0923@hotmail.com; 4 School of Chinese Medical Sciences, Henan University of Chinese Medicine, Zhengzhou 450046, China

**Keywords:** RuHaoDaShi granules, influenza A (H1N1) virus, network pharmacology, ferroptosis

## Abstract

Objective: This study aims to investigate the pharmacodynamic effects and underlying mechanisms of the Chinese herbal formula RuHaoDaShi (RHDS) granules against the influenza virus in experimental models. Methods: This study aims to employ network pharmacology to identify the active components of RHDS and its potential targets and mechanisms of action against H1N1. The molecular docking approach validated the interactions between the core targets and the RHDS compounds. In vitro, the antiviral activity of RHDS was assessed by therapeutic, prophylactic, and premixed administration to H1N1-infected A549 cells. An in vivo experiment was conducted using a mouse H1N1 pneumonia model. The model was treated with a dose of 1.04, 2.08, and 4.16 g/kg of RHDS, administered via gavage daily. The study’s objective was to evaluate the antiviral activity and mechanism of action of RHDS in mice. Mice were evaluated on day 6 by assessing survival, viral load (RT-qPCR), lung pathology (HE staining), inflammatory cytokines (ELISA, immunohistochemistry), and ferroptosis markers (WB, qPCR). Results: Network pharmacology identified 77 biologically active RHDS compounds (e.g., quercetin and kaempferol) and 32 core targets common to RHDS, H1N1, and ferroptosis. Molecular docking was used to verify a high affinity for binding between the core targets HIF-1α, MAPK3, and key RHDS compounds. In vitro studies demonstrated that RHDS exhibited protective properties against H1N1-infected cells, with the therapeutic delivery method proving the most efficacious. In vivo studies have shown that RHDS reduces mortality, lung index, and viral load in mice while attenuating histopathological damage. The study demonstrated a reduction in the release of inflammatory cytokines, including IL-6, IFN-γ, and IL-17A, and decreased expression levels of MPO and F4/80 proteins in lung tissue. Mechanistically, the administration of RHDS resulted in the up-regulation of the expression levels of GPX4, SLC7A11, and Nrf2 proteins while concomitantly inhibiting the expression of HIF-1α, COX2, and ACSL4. These findings confirm the modulatory effect of RHDS on the GPX4/SLC7A11/Nrf2 pathway. Conclusions: RHDS demonstrated a protective effect against H1N1-induced cytopathy in vitro and was effective in attenuating H1N1-induced pneumonia in murine models. The study suggests that RHDS has antiviral potential to treat H1N1 viral pneumonia by modulating inflammatory cytokines and the GPX4/SLC7A11/Nrf2 pathway.

## 1. Introduction

Influenza is an acute viral respiratory disease that poses a significant global health challenge. The World Health Organization (WHO) estimates that approximately one billion individuals are infected with the influenza virus each year, resulting in an annual death toll of between 290,000 and 650,000 [1]. Influenza viruses are classified into four genera based on antigenic differences in their nucleoprotein (NP) and matrix protein (M). Among these genera, Influenza A virus (IAV) has the most significant clinical significance due to the epidemics it causes yearly in populations. This zoonotic pathogen has the potential to be transmitted among a variety of bird and mammal species [2]. There have been occasions when certain events have triggered pandemic outbreaks. Notable examples include the Spanish flu of 1918, the Asian flu of 1957, the Hong Kong flu of 1968, and the H1N1 flu outbreak in the United States in 2009. These events have imposed a significant economic and healthcare burden on affected regions [3]. The H1N1 virus is a specific type of IAV that results in upper and lower respiratory tract infections in infected hosts. The symptoms associated with this condition include chills, fever, cough, and pneumonia. In severe cases, the condition can lead to death [4]. IAVs are known to evolve through a process involving reclassification and mutation. It is imperative to consider the potential ramifications of this evolutionary trajectory on the host specificity and pathogenicity of these viruses, as it could potentially culminate in a human pandemic [5,6]. Furthermore, the considerable variability of IAV results in the apparent limitation of the effectiveness and coverage of the vaccine [7]. Consequently, antiviral medications remain the primary treatment option. Only Three drug classes have been approved by the U.S. Food and Drug Administration (FDA): Non-steroidal anti-inflammatory drugs (NSAIDs), M2 ion channel blockers and the PA endonuclease inhibitor [8]. Despite the efficacy of these antiviral medications in suppressing viral replication, the high degree of variability in IAV has resulted in the rapid emergence of drug resistance. For instance, the FDA no longer recommends the use of M2 blockers for the treatment of IAV [9]. While most pandemic influenza virus strains remain susceptible to NA inhibitors, developing resistance remains probable, as evidenced by the experience with M2 inhibitors. For instance, the H275Y mutation that emerged during the 2009 pandemic H1N1pdm09 strain resulted in the development of oseltamivir resistance [10]. This concern has driven the development of novel influenza antivirals with higher barriers to resistance.

Ferroptosis is a regulated form of cell death characterized by the accumulation of lipid-reactive oxygen species (ROS) in the presence of iron [11]. The virus disrupts the body’s intracellular iron and redox homeostasis during an infection. This disruption has the potential to increase either intracellular iron concentrations or intestinal iron absorption, either of which may subsequently induce ferroptosis [12,13]. Research has demonstrated that IAV hemagglutinin (HA) facilitates viral replication by inducing cellular ferroptosis via ferritin autophagy, leading to lipid peroxidation and impairing the MAVS-mediated type I IFN response [14]. As a selenium-dependent antioxidant enzyme and critical regulator of ferroptosis, glutathione peroxidase 4 (GPX4) constitutes a central node in the antioxidant defense axis [15]. Dysregulation of core components in this axis—particularly solute carrier family 7 member 11 (SLC7A11) and GPX4—compromises cellular antioxidant capacity, thereby triggering ferroptosis [16]. Furthermore, nuclear factor erythroid 2-related factor 2 (Nrf2), which transcriptionally regulates GPX4, serves as a pivotal upstream modulator of the antioxidant stress response [17]. The GPX4/SLC7A11/Nrf2 pathway is a predominant mechanistic framework in the domain of ferroptosis research, exerting a regulatory role over cellular redox homeostasis. However, research on pharmaceutical ferroptosis inhibition for H1N1 treatment remains limited, underscoring its potential as a promising therapeutic target for H1N1 antivirals.

**Figure 1:**
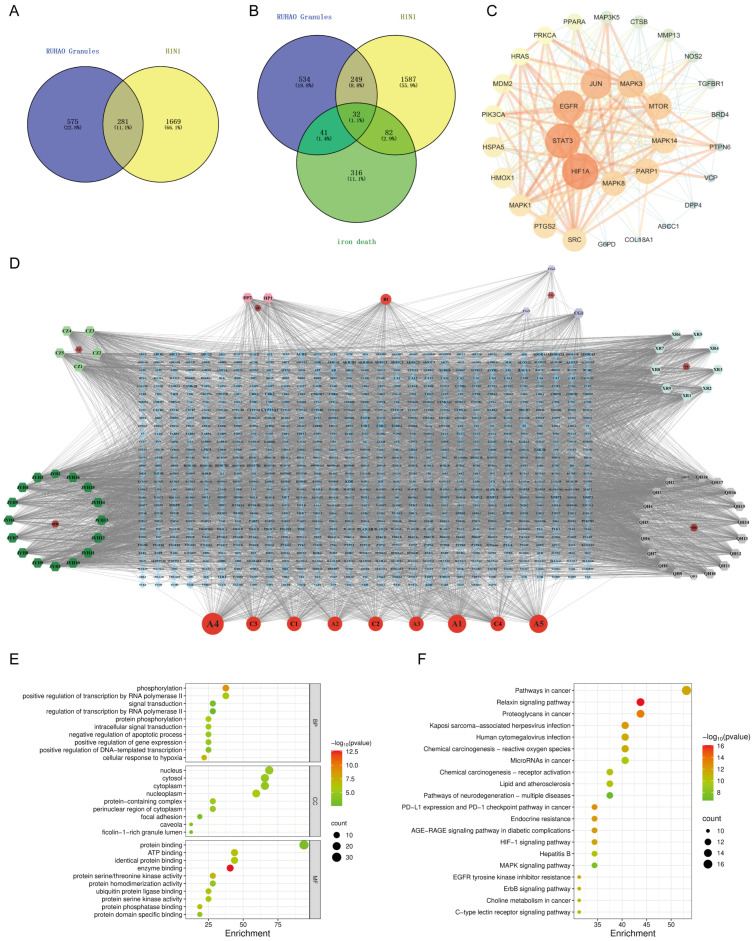
Network pharmacology analysis results of RHDS granules: (**A**) RHDS and H1N1 intersecting targets; (**B**) RHDS and H1N1 and ferroptosis intersecting targets; (**C**) PPI network diagram of core targets; (**D**) “RHDS compound–target” network of RHDS granules; (**E**) GO enrichment analysis diagram; (**F**) KEGG pathway enrichment analysis bubble diagram.

Chinese medicine boasts several distinctive advantages in the treatment of respiratory viruses. The elements mentioned above comprise synergistic effects, minimal side effects, low drug resistance, and especially immunomodulation and other multi-component and multi-target features [18,19]. Influenza disease, with its rapid onset, rapid transmission, and wide range of infected people, belongs to the category of “*warm and hot disease*” and “*warm and epidemic disease*” in traditional Chinese medicine (TCM). It is evident that Chinese medicine has a distinctive role and benefit in the treatment of viral diseases, as evidenced by its long-standing application in the management of various ailments, including “*The Treatise on Typhoid Fever*” dating back 1800 years, as well as the recent clinical practices involving atypical pneumonia (SARS), avian influenza, and new coronavirus pneumonia. Extensive evidence demonstrates that traditional Chinese medicine (TCM) inhibits viral replication through multi-faceted interference with the viral life cycle [20]. Furthermore, TCM modulates inflammatory responses, mitigating cytokine storm-mediated acute lung injury. Additionally, specific TCM preparations have been observed to possess robust inhibitory activity against the renin–angiotensin system (RAS) [21]. Moreover, the multi-pathway and multi-target pharmacodynamic advantages of TCM enable it to alleviate acute lung injury caused by influenza and reduce disease duration [22]. RuHaoDaShi granules (RHDS) represent the clinical experience formula developed by Professor Haitong Wan of Zhejiang Chinese Medicine University. In clinical trials, the granules demonstrated efficacy in the treatment of influenza. Consequently, research efforts are now focused on developing novel TCM-derived anti-influenza agents. In the formula, *Elsholtzia ciliata* and *Artemisia carvifolia* are used as the monarch herbs, while *Lonicera japonica* and *Houpoea officinalis* are used as the ministerial herbs, and *Atractylodes Lancea* and *Amomum tsaoko* are used as the adjuvant herbs. As documented in the literature, all six herbs exhibit anti-inflammatory, antibacterial, and antiviral properties. *Elsholtzia ciliata* has been shown to impact all phases of the viral life cycle, exhibiting a comprehensive antiviral profile. Most of its constituents possess mechanisms that impede the NF-κB and MAPK signaling pathways [23,24]. *Lonicera japonica* and its active ingredients have various pharmacological effects, such as antibacterial, anti-inflammatory, antiviral, and antitumor effects. They also play an essential role in preventing and treating serious human and zoonotic toxic diseases [25,26,27]. The present study demonstrates that *Atractylodes Lancea* extract significantly attenuates IAV-induced lung injury by modulating the TLR7 signaling pathway [28,29]. A preliminary investigation of RHDS in the group’s initial phase revealed that it possesses antipyretic and anti-inflammatory properties. However, the pharmacodynamic effects and specific mechanisms by which it treats H1N1 remain to be elucidated through further research. The present study addresses this research gap by experimentally validating the antiviral effects and specific mechanisms of action of RHDS in vivo and in vitro. The advent of network pharmacology has engendered novel concepts and methodologies for investigating the therapeutic mechanisms of RHDS. Network pharmacology is a systematic approach that analyzes the interrelationship between drug components and disease targets, thereby unveiling the multi-component and multi-target mechanism of drug action. In this study, we employed network pharmacology and molecular docking technology to conduct a preliminary investigation into the active ingredients, targets, and signaling pathways of RHDS against the influenza A virus. We also investigated the antiviral activity of RHDS in vitro and in vivo using experiments to assess its efficacy against influenza. Given that GPX4/SLC7A11/Nrf2 is the master regulator of ferroptosis, and our network pharmacology prediction identified HIF-1α as a key upstream target of RHDS (Figure 1), we prioritized it as a mechanistic focus to integrate viral cytopathy, inflammation, and cell death.

## 2. Materials and Methods

### 2.1. Materials and Reagents

RuHaoDaShi granules, overseen by the project team’s preparation center (batch no.220202) [30]. The positive control drug was oseltamivir phosphate capsules from Zhongshan Wanhan Pharmaceutical Co., Ltd. (182D1110, Zhongshan, China). QingReJieDu oral liquid was from Hubei Taizi Pharmaceutical Co., Ltd. (20230818, Jingmen, China). Fetal bovine serum (FBS) was purchased from GIBCO BRL (10270-106, Grand Island, NY, USA); penicillin–streptomycin solution was purchased from Shanghai Macklin Biochemical Technology (P917928, Shanghai, China); F12k medium was purchased from Boster Biological Technology (PYG0036, Wuhan, China); mouse IL-2, IL-6, IL-17A and IFN-γ ELISA kits were purchased from Jiangsu Meibiao Biotechnology Co., Ltd. (Yancheng, China); BCA protein assay kit was purchased from Beyotime Biotechnology (P0012, Shanghai, China); CCK-8 kit was purchased from Beijing Zoman Biotechnology (zp328-2, Beijing, China); 3% BSA was purchased from Shanghai yuanye Bio-Technology (R27679, Shanghai, China); FastPure Cell/Tissue Total RNA Isolation Kit V2 was purchased from Vazyme Biotech (RC112-01, Nanjing, China); PrimeScript™ RT Master Mix was purchased from Takara (RR036A, Tokyo, Japan); DAB was purchased from Fuzhou Maixin Biotech (DAB-1031, Fuzhou, China); Primary Antibody MPO (22225-1-AP) and F4/80 (27044-1-AP) were purchased from Proteintech Group (Wuhan, China); primary antibodies GPX4 (ab125066), SLC7A11 (ab307601), ACSL4 (ab155282), GAPDH (ab181602) and β-actin (ab8226) were purchased from Abcam (Cambridge, MA, USA); HIF-1α (CST 36169) and COX2 (CST 36169) were purchased from Cell Signaling Technology (Danvers, MA, USA); Nrf2 (PA5-88084), Goat anti Nrf2 (PA5-88084), Goat anti-Mouse IgG(H+L) secondary antibody (31160), and Goat anti-Rabbit IgG(H+L) secondary antibody (31210) were purchased from Thermo Fisher Scientific (Waltham, MA, USA); and secondary antibodies were purchased from Cell Signaling Technology (Danvers, MA, USA). HRP-labeled IgG secondary antibody was purchased from Beijing Zhongshan Golden Bridge Biotechnology (PV6001/6002, Beijing, China).

### 2.2. Cells, Viruses, and Animals

A549 cells were preserved in the College of Chinese Medicine for Cardiovascular-Cranial Disease, Zhejiang Chinese Medical University (ZCMU) and cultured in the F12k medium (Boster Biological Technology, Wuhan, China) containing 10% FBS (Gibco, NY, USA) and 1% penicillin–streptomycin (Macklin, Shanghai, China) in the F12k medium at 37 °C in a CO_2_ incubator with 5% CO_2_.

The standard virus strain of influenza A virus A/PR/8/34 was provided by the Zhejiang Provincial Center for Disease Control and Prevention and stored in a refrigerator at −80 °C. Before utilization, the viral titer TCID_50_ was determined to be within the range of 10^4.5^/0.1 mL, as substantiated by the 50% tissue culture infectious dose (TCID_50_) calculation, employing the Reed-Muench method.

The experimental animals were male SPF grade BALB/c mice (16 ± 1 g) and SD rats (250–280 g) at 6–8 weeks of age. The animals were housed in the ABSL-2 laboratory of Zhejiang University Center for Veterinary Sciences. Temperature was controlled at 22–25 °C, relative humidity was maintained at 50–60%, and a 12 h light/dark cycle was employed. The animals were provided with ad libitum access to food and water. The animals were provided by the Zhejiang University Center for Veterinary Sciences (Ethical Approval No ZJU20230478) and were used for the experiments after one week of acclimatization. This period of acclimatization was intended to ensure the stability of the animals and to reduce the influence of individual differences on the experimental results.

### 2.3. Network Pharmacology Analysis

The pharmacological targets of RHDS were systematically screened employing a network pharmacology approach. The active ingredients of the constituents of RHDS—namely, *Elsholtzia ciliata*, *Artemisia carvifolia*, *Lonicera japonica*, *Houpoea officinalis*, *Atractylodes Lancea*, and *Amomum tsaoko*—were identified through a screening process based on the criteria of oral bioavailability (OB) ≥ 30% and drug-likeness (DL) ≥ 0.18. This screening process was conducted using the TCMSP database. SwissADME then employed the molecular structures and SMILES numbers that PubChem has validated to predict the target of a given component. This prediction was used to construct a network that illustrates the interactions between the component and its target. The target genes for H1N1 and ferroptosis were retrieved from GeneCards, OMIM, and FerrDB databases. Intersecting targets between RHDS, H1N1, and ferroptosis were identified and visualized by Venny 2.1.0. The screened intersecting targets were then analyzed using STRING 12.0 and Cytoscape 3.10.2 to generate a protein–protein interaction (PPI) network and to prioritize the top-ranked core targets (degree ≥ 10). The “RHDS component–target” network was constructed. A comprehensive investigation was conducted to ascertain the biological processes, molecular functions, cellular components, and signaling pathways associated with RHDS. This investigation involved implementing functional enrichment analysis, encompassing both Gene Ontology (GO) and Kyoto Encyclopedia of Genes and Genomes (KEGG) pathways. The analysis was conducted using Metascape, a comprehensive data analysis platform, to elucidate the underlying mechanisms and pathways involved. The interactions between top-ranked targets and RHDS core compounds were validated through molecular docking with AutoDock Vina 1.1.2, and binding patterns were subsequently visualized using PyMOL and LigPlot+. Docking affinity ≤ −5 kcal/mol validated target interactions.

### 2.4. Preparation of Drug-Containing Serum for RHDS

All SPF-grade healthy male SD rats (*n* = 24) were acclimatized, fed for 7 days, and divided into control and RHDS low-, medium-, and high-dose groups (0.72, 1.44, and 2.88 g/kg/d, n = 6/group). The drug was administered by gavage twice daily for 7 days, and the control group was given the same dose of saline. The murine dosages of Ruhodashi granules were uniformly determined through body surface area (BSA)-based species scaling, using the human-to-mouse conversion factor of 6.3 calibrated against their respective clinical doses and the standard human body weight of 70 kg. The rats were fasted for 12 h before the last administration, and blood was collected by abdominal aortic puncture 1 h after the administration. Serum was separated by centrifugation at 3000× *g* for 15 min, inactivated at 56 °C for 30 min, filtered through Millipore syringe filters of 0.22 μm (SLGP033R, Merck Millipore, Billerica, MA, USA), and freeze-stored at −80 °C for backup.

### 2.5. RHDS Cytotoxicity Assay

The RHDS-containing sera from the low, medium, and high groups were serially diluted to eight concentrations (5%, 10%, 15%, 20%, 25%, 30%, 35%, and 40%) in F12K medium. Control serum was diluted to match drug–serum concentrations. A CCK-8 assay was performed on the A549 cells to detect the cytotoxic effect of RHDS. The cells were cultivated in 96-well plates at a 1 × 10^5^/mL density and subsequently incubated in a cell culture incubator for 24 h. Following the removal of the medium, the adherent cells were washed twice with phosphate-buffered saline (PBS). Then, different concentrations of drug-containing serum were added according to the grouping in order (100 μL/well, n = 6 for each concentration). Serum-free F12k medium (100 μL/well) was utilized for the control group. Following a 24 h incubation period, 10 μL of CCK-8 reagent was added to each well and incubated at 37 °C in a light-protected environment for 1 h. The absorbances were subsequently measured at 450 nm using an enzyme marker (Infinite^®^ 200 PRO, Tecan, Männedorf, Switzerland).

### 2.6. RHDS In Vitro Anti-H1N1 Activity Assay

A549 cells were inoculated into 96-well plates and incubated at 37 °C and 5% CO_2_ for 24 h. Subsequently, different dose groups of RHDS-containing sera were inoculated into cell plates at the maximum nontoxic concentration, with six replicate wells established for each concentration gradient. To administer therapeutic agents, the cells were infected with 100 TCID_50_ H1N1 (100 μL/well, adsorbed for 2 h). Following the removal of the medium, the cells were treated with the maximum nontoxic concentration of RHDS-containing serum from the low-, medium-, and high-dose groups, as well as with the maximum nontoxic concentration of the positive control group (oseltamivir and QRJD oral solution). Cell survival was assessed by incubating the samples for 24 h after infection. For premixed administration, 100 TCID_50_ H1N1 was mixed with the maximum nontoxic concentration of drug-containing serum for 2 h at 4 °C, then added to the cells and incubated for 2 h at 37 °C. The replacement of the viral maintenance medium followed this. The superior portion of the tissue was then discarded and washed with phosphate-buffered saline (PBS). Subsequently, the cells were subjected to an incubation period at 37 °C for 24 h to assess cell viability comprehensively. To administer prophylactically, the cells were pre-treated with the maximum nontoxic concentration of RHDS-containing serum in the low-, medium-, and high-dose groups, as well as the maximum nontoxic concentration in the positive control group (oseltamivir and QRJD oral solution) for 2 h. The cells were washed with PBS and then infected with 100 TCID_50_ H1N1 (100 μL/well, adsorbed for 2 h). Following the removal of the medium, the culture was transitioned to a serum-free medium and maintained for 24 h to facilitate an assessment of cell viability.

### 2.7. RHDS In Vivo Anti-H1N1 Activity Assay

The BALB/c mice were randomly divided into eight groups of 10 mice each. The groups included a control group, a model group, and low-, medium-, and high-dose groups of RHDS (1.04, 2.08, and 4.16 g/kg/d), respectively. Additionally, the study included an oseltamivir group (19.5 mg/kg/d) and QingReJieDu oral liquid group (3.9 mL/kg/d). The mice were randomized via a random number table. Drug administrators and outcome assessors were blinded to group assignments. The murine dosages of oseltamivir, QingReJieDu oral liquid, and Ruhodashi granules were uniformly determined through body surface area (BSA)-based species scaling, using the human-to-mouse conversion factor of 9.1 calibrated against their respective clinical doses and the standard human body weight of 70 kg. The control and model groups were administered an equivalent volume of saline solution. Following a five-day acclimatization period, the mice were anesthetized with sodium pentobarbital and infected with 50 μL of the virus, 10 LD_50_. The drugs were administered via gavage five days after influenza virus infection according to the group. Body weight and mortality were recorded daily. Following the previous administration, blood was collected via the fundus plexus vein method, weighed, and analyzed. The organ tissues were then dissected and removed, rinsed with saline, weighed after filter paper absorbed the water, and the organ index was calculated. The protective effect of RHDS was evaluated based on the change in body weight of mice with organ index.

### 2.8. Hematoxylin and Eosin (HE) Staining

The lung tissues of mice in each group were fixed in 4% paraformaldehyde for 24 h, embedded in paraffin, and sectioned at a thickness of 4–6 μm. After xylene deparaffinization and an ethanol series rehydration, the sections underwent hematoxylin and eosin (HE) staining. The sections were subjected to HE staining. Pathologic changes were examined using a microscope.

### 2.9. Immunohistochemical Staining

The protein expression levels of MPO and F4/80 in mouse lung tissues were examined using immunohistochemistry, and 4–6 μm paraffin sections were incubated for 10 min (3% H_2_O_2_) in citrate buffer for antigen repair, followed by endogenous peroxidase blocking. After being incubated with 3% BSA (Shanghai, China) for 1 h, the sections were incubated with primary antibodies MPO (Proteintech, Wuhan, China) (1:500 dilution) and F480 (Proteintech, Wuhan, China) (1:1000 dilution) were co-incubated for 1.5 h, followed by incubation with HRP-labeled IgG secondary antibody (ZSGB-BIO, Beijing, China) for 30 min at room temperature. Then, DAB (Fuzhou Maixin Biotech, Fuzhou, China) color development reaction was performed. The images were observed and captured under a microscope. Quantitative analysis of positively stained areas was performed using the ImageJ software (version 1.54g).

### 2.10. Elisa Assay

The levels of IL-2, IL-6, IL-17A, and IFN-γ in mouse serum were measured by ELISA kits. The experimental methods were carried out by the manufacturer’s protocol, as stipulated by Jiangsu Meibiao Biological Technology Co., Ltd. (Jiangsu, China). The standards and sera of each group (50 μL/well) were incubated in plates pre-coated with HRP-conjugated detection antibody for 60 min. Following the completion of the washing step, the color development stage was initiated using a TMB substrate. The samples were then incubated for 15 min, during which they were protected from light exposure. The termination solution was then applied, and the process was terminated. The degree of colouration was subsequently measured by reading the sample’s absorbance at a wavelength of 450 nanometers. This measurement was taken within 30 min using an enzyme marker (Infinite^®^ 200 PRO, Tecan, Männedorf, Switzerland).

### 2.11. RT-qPCR Analysis

The RNA from each group of lung tissues was extracted using the FastPure Cell/Tissue Total RNA Isolation Kit V2 (Vazyme, Nanjing, China). The concentration and purity of the RNA were then determined by a microRNA protein analyzer (Nanodrop one, Thermo Fisher Scientific, Waltham, MA, USA). Subsequently, reverse transcription was performed using a reverse transcription kit (Takara, Tokyo, Japan). The target and internal reference genes were amplified by fluorescence quantitative PCR according to the protocol provided by the manufacturer (Yeasen Biotechnology Co., Ltd., Shanghai, China). The mRNA expression levels of the genes were calculated using the 2 (^−ΔΔ^Ct) method. The primers were procured from Sangon Biotech, a company based in Shanghai, China. The sequences are presented in Table 1.

### 2.12. Western Blot Analysis

The expression levels of GPX4, SLC7A11, NRF2, COX2, ACSL4, and HIF-1α proteins in lung tissues were detected by Western blot. Lung tissues from each group were obtained and homogenized in pre-cooled RIPA lysis buffer (Thermo Fisher Scientific, Waltham, MA, USA) using an electric homogeniser (Shanghai, China). The resulting mixture was then centrifuged (12,000× *g*, 10 min, 4 °C) to collect the upper layer. The protein concentration was determined and normalized using a BCA kit (Beyotime, Shanghai, China). The separation of proteins was achieved through SDS-PAGE electrophoresis, which involved preparing a system comprising a 10% separating gel and a 5% concentrating gel, with a total loading volume of 30 μg of protein per well. After the completion of the electrophoresis, a wet transfer was conducted for 1.5 h at a constant current of 200 milliamperes. This transfer was facilitated by a polyvinylidene fluoride (PVDF) membrane obtained from Merck Millipore, a company based in Billerica, Massachusetts. The membranes were closed for 1 h at room temperature after transferring using a rapid closure solution. They were subsequently incubated with primary antibodies GPX4 (1:1000), SLC7A11 (1:1000), Nrf2 (1:500), COX2 (1:1000), ACSL4 (1:20,000), and HIF-1α (1:1000), respectively, overnight at 4 °C. GAPDH (1:10,000) and β-actin (1:2000) were utilized as internal reference proteins. On the following day, the cells were washed thrice (10 min each) with TBST (Tris-buffered saline containing 0.1% Tween-20), followed by incubation with the corresponding secondary antibodies: goat anti-mouse IgG (H+L) secondary antibody (1:5000) and goat anti-rabbit IgG (H+L). The membrane was subjected to pretreatment with ECL DualVue WB Marker (Merck Millipore, Billerica, MA, USA), followed by a series of five washes with TBST, before the detection stage of the experiment. The image analysis was performed using the ImageJ software version 1.48.

### 2.13. Statistical Analysis

The experimental results were analyzed using GraphPad Prism 9.5.0, and the experimental data were expressed as the mean ± standard deviation (x ± s). One-way ANOVA-Tukey’s multiple comparisons test was used to analyze the experimental data to analyze the ANOVA between multiple groups, and *t*-tests between two groups (tests and nonparametric tests) assessed significant differences. Tukey’s test was applied for all multi-group comparisons to correct Type I error inflation. A *p*-value less than 0.05 was considered statistically significant.

## 3. Results

### 3.1. Results of Network Pharmacology Analysis

**Figure 2:**
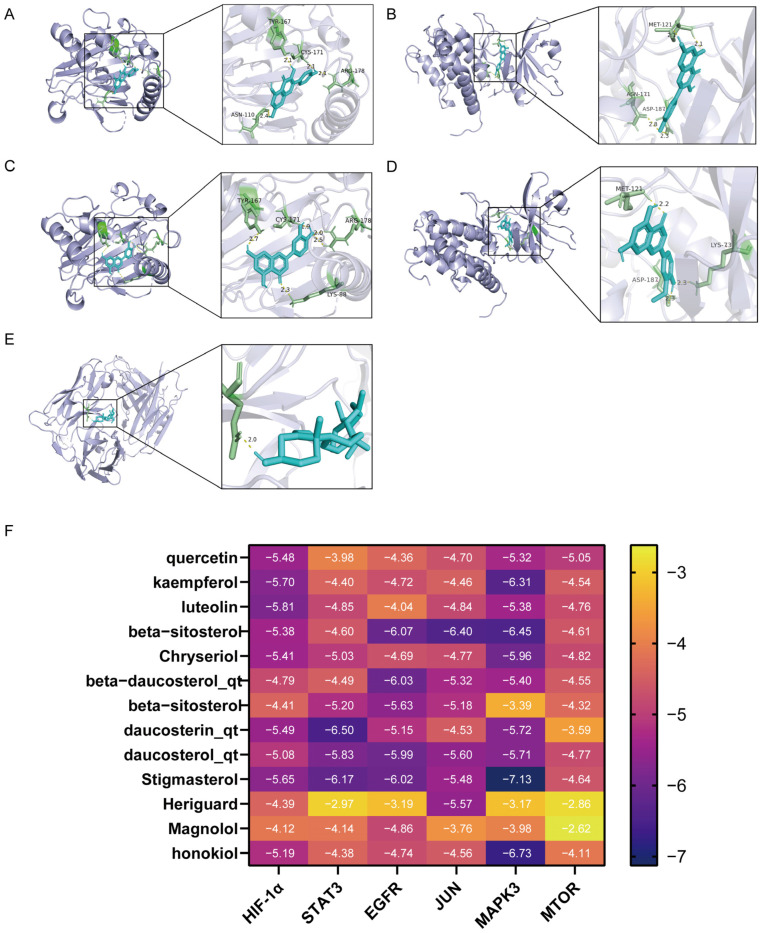
Diagram of molecular docking modeling of key RHDS granules with receptor proteins: (**A**) HIF-1α-kaempferol (−5.70 kcal/mol); (**B**) MAPK3-kaempferol (−6.31 kcal/mol); (**C**) HIF-1α-luteolin (−5.81 kcal/mol); (**D**) MAPK3-Chryseriol (−5.96 kcal/mol); (**E**) EGFR-beta-beta- sitosterol 3-O-glucoside_qt (−6.07 kcal/mol); (**F**) molecular docking binding heat map (kcal/mol).

The network analysis identified 77 active compounds from the RHDS component, with 865 predicted targets. The results of the cross-tabulation analyses (Figure 1A,B) indicated that there were 32 common targets shared by RHDS, H1N1 (1950 targets), and ferroptosis (649 targets). The protein–protein interaction (PPI) network of these 32 targets demonstrated that HIF-1α, STAT3, Epidermal Growth Factor Receptor, JUN, and MAPK3 were the highest-ranked hubs (degree ≥ 14.9) (Figure 1C). The “RHDS compound–target” network (Figure 1D) ranked quercetin (MOL000098), kaempferol (MOL000422), and lignocerol (MOL000006) as core bioactive components. The GO enrichment analysis (Figure 1E) indicated that RHDS is implicated in several biological processes, including phosphorylation, transcriptional regulation, nuclear/cytoplasmic localisation, and protein/ATP binding. The KEGG enrichment analysis further revealed that the mechanism of RHDS for H1N1 treatment may be significantly associated with signaling pathways such as the relaxin signaling pathway. As demonstrated in Figure 2, molecular docking analysis substantiated the substantial binding (affinity < −5 kcal/mol) between the pivotal targets HIF-1α, MAPK3, and the primary compounds of RHDS, kaempferol, and lignocerol. HIF-1α-kaempferol (−5.70 kcal/mol) and MAPK3-kaempferol (−6.31 kcal/mol) were the most pronounced interactions. The first subject demonstrated the optimal interaction. These findings suggest that RHDS may combat H1N1 through multi-targeted modulation of viral replication, iron metabolism, and inflammation-related pathways.

### 3.2. Analysis of the In Vitro Anti-H1N1 Activity of RHDS

**Figure 3:**
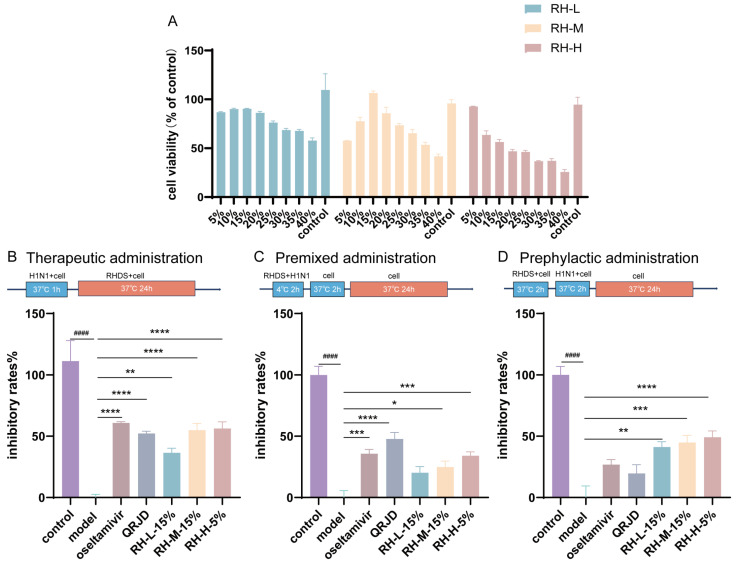
The present study investigates the cytotoxic effects of RHDS-containing serum on A549 cells and the anti-H1N1 properties of RHDS when administered in three distinct ways: (**A**) toxicity injury in the A549 cells was assessed using the CCK-8 assay after treatment with increasing concentrations of RHDS-containing serum for 24 h. (**B**) Therapeutic administration; (**C**) premixed administration; (**D**) prephylactic administration. The above data are expressed as means ± SD. (*n* = 6); ^####^ *p* < 0.0001 vs. control group; * *p* < 0.05, ** *p* < 0.01, *** *p* < 0.001, and **** *p* < 0.0001 vs. model group.

In order to ascertain the inhibitory effect of RHDS on influenza A virus, the maximum nontoxic concentration of RHDS low-, medium-, and high-dose-containing serum on cells was examined. The CCK-8 method was used to assess the safety of RHDS and to determine the dose for subsequent experiments. The results (Figure 3A) demonstrated that RHDS had minimal toxic effects on cells. No cytotoxicity was observed. According to the results, 15% low-dose, 15% medium-dose, and 5% high-dose RHDS-containing serum were used as the maximum nontoxic concentration of RHDS for subsequent experiments, respectively. The anti-H1N1 activity of RHDS was determined by testing the inhibition of the influenza virus by different modes of administration. As illustrated in Figure 3B, the therapeutic dosing group exhibited a substantially higher viral inhibition rate compared to the model group for RH-M (15%), RH-H (5%), and oseltamivir. The oseltamivir group had the highest viral inhibition rate among these groups, followed by RH-H (5%). In Figure 3C, the premixed dosing group demonstrated a heightened viral inhibition rate for RH-H (5%) and oseltamivir. Figure 3D shows that RH-L (15%), RH-M (15%), and RH-H (5%) in the prophylactic dosing group exhibited a higher viral inhibition rate. Meanwhile, the viral inhibition rates of oseltamivir and QRJD did not differ significantly. It has been demonstrated that RHDS-containing serum can exert anti-H1N1 effects through three pathways of treatment: therapeutic administration, premixed administration, and prephylactic administration. However, the most significant impact is observed with therapeutic administration.

### 3.3. Protective Effects of RHDS in H1N1-Infected Mice

**Figure 4:**
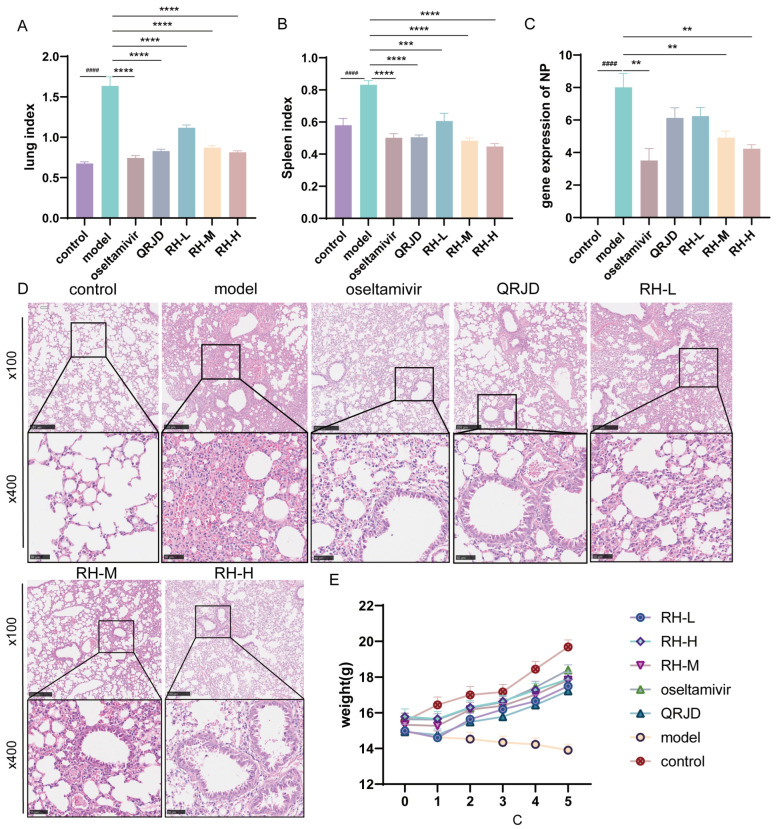
Antiviral effects of RHDS on H1N1-infected mice: (**A**) lung index of mice in each group; (**B**) splenic index of mice in each group; (**C**) expression level of viral NP gene, (n = 6); (**D**) HE staining of lung tissues in each group, (n = 6) (scale bar: 250 μm, 50 μm); (**E**) trend of body weight change in mice in each group. The above data are expressed as means ± SD; ^####^ *p* < 0.0001 vs. control group; ** *p* < 0.01, *** *p* < 0.001, and **** *p* < 0.0001 vs. model group.

To investigate the protective effect of RHDS on H1N1-infected mice, we conducted in vivo animal experiments. During the experimental period, the mice in the control group exhibited good mental status, sensitive activity, smooth fur, and stable weight growth (Figure 4E). Fur coat loss and significant weight decrease (Figure 4E). These symptoms are consistent with those of a viral infection. Lung index analysis (Figure 4A) demonstrated that the extent of lung tissue edema was considerably higher in the model group (*p* < 0.0001). The RHDS intervention exhibited a dose-dependent effect in mitigating these pathological processes. In comparison with the model group, the mice in the RH-M, the RH-H, and the oseltamivir group exhibited a significant increase in body weight (Figure 4E). Furthermore, a concurrent enhancement in the lung index and the splenic index was observed (Figure 4A,B). The NP is a structural protein of influenza viruses that is critical for viral infection and replication [31]. The NP gene, being relatively conserved and specific among influenza viruses, can indicate viral load by measuring its mRNA expression level. To observe the changes in viral load after RHDS administration, the mRNA expression level of NP in the lung tissues of mice in each group was detected using RT-qPCR. The results demonstrated (Figure 4C) that the mRNA expression level of NP was considerably higher in the model group compared with the control group, indicating an augmented viral load (*p* < 0.0001). Furthermore, the mRNA expression level of NP was notably lower in the RH-L, RH-M, and RH-H groups compared with the model group (*p* < 0.01 or *p* < 0.0001). Among the study’s participants, viral load reduction was most significant in the RH-H and oseltamivir groups. Lung tissue histopathological analysis further confirmed the efficacy of the RHDS intervention, and hematoxylin–eosin (HE) staining revealed that the alveolar structure was compromised in the model group, accompanied by substantial inflammatory infiltration and areas of solid lesions. The RH-H group exhibited a marked suppression of the inflammatory response, characterized by a decrease in inflammatory cell infiltration and a reduction in the area of lesions (Figure 4D). The results, as mentioned above, indicated that RHDS could alleviate the lung injury caused by H1N1 infection through the mechanisms of reducing the viral load of lung tissues, attenuating the inflammatory infiltration, and improving tissue edema.

### 3.4. Inhibition of H1N1 Cytokines by RHDS

**Figure 5:**
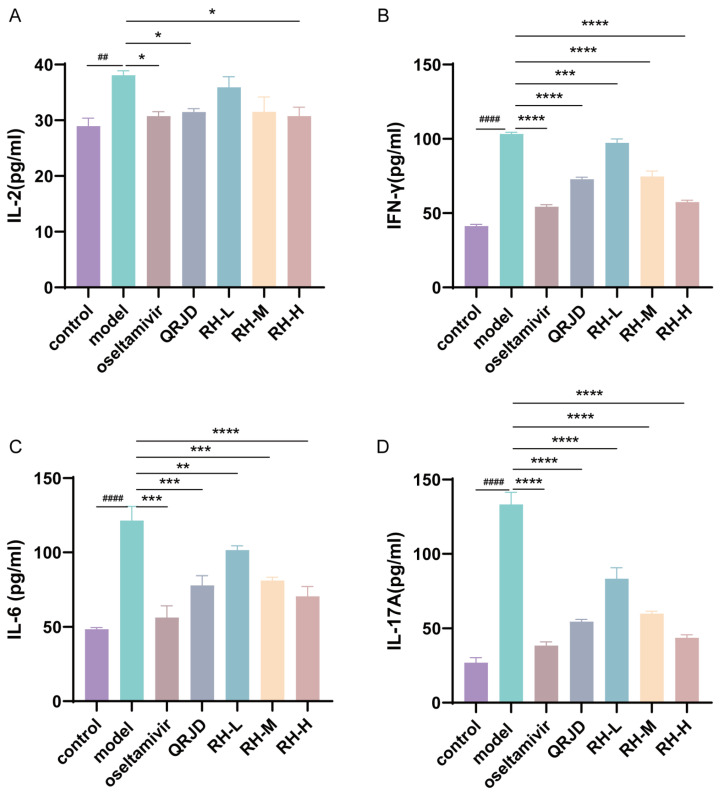
In vivo effects of RHDS on H1N1-induced cytokines IL-2, IL-6, IL-17A, and IFN-γ. Levels of cytokines present in the serum of each group of mice were detected using ELISA kits: (**A**) IL-2, (**B**) IFN-γ, (**C**) IL-6, (**D**) IL-17A. The above data are expressed as means ± SD. (n = 6); ^##^ *p* < 0.01, ^####^ *p* < 0.0001 vs. control group; * *p* < 0.05, ** *p* < 0.01, *** *p* < 0.001, and **** *p* < 0.0001 vs. model group.

The expression levels of cytokines in mouse serum were detected using ELISA kits. IL-2, IL-6, IL-17A, and IFN-γ are common immune factors in viral infections. These factors prompt immune cells to accumulate at the site of inflammation, exacerbating inflammatory reactions and leading to cellular infiltration and tissue damage at the site of inflammation. The experimental results demonstrated that H1N1 infection significantly increased the expression of pro-inflammatory factors, including IL-2, IL-6, IL-17A, and IFN-γ, in comparison with the control group (Figure 5). In contrast to the model group, the RH-L, RH-M, and RH-H groups exhibited a substantial decrease in the expression level of IFN-γ and IL-6 (*p* < 0.0001-*p* < 0.01). The study’s results demonstrated a significant reduction in the expression level of IL-2 in the RH-H group (*p* < 0.05). In contrast, the RH-L, M, and H groups exhibited a dose-dependent down-regulation of IL-17A (*p* < 0.0001). These results suggest that the administration of RHDS therapy can effectively attenuate the excessive immune response triggered by H1N1 infection.

### 3.5. RHDS Reduces Protein Expression Levels of MPO and F4/80 in Lung Tissues of H1N1-Infected Mice

**Figure 6:**
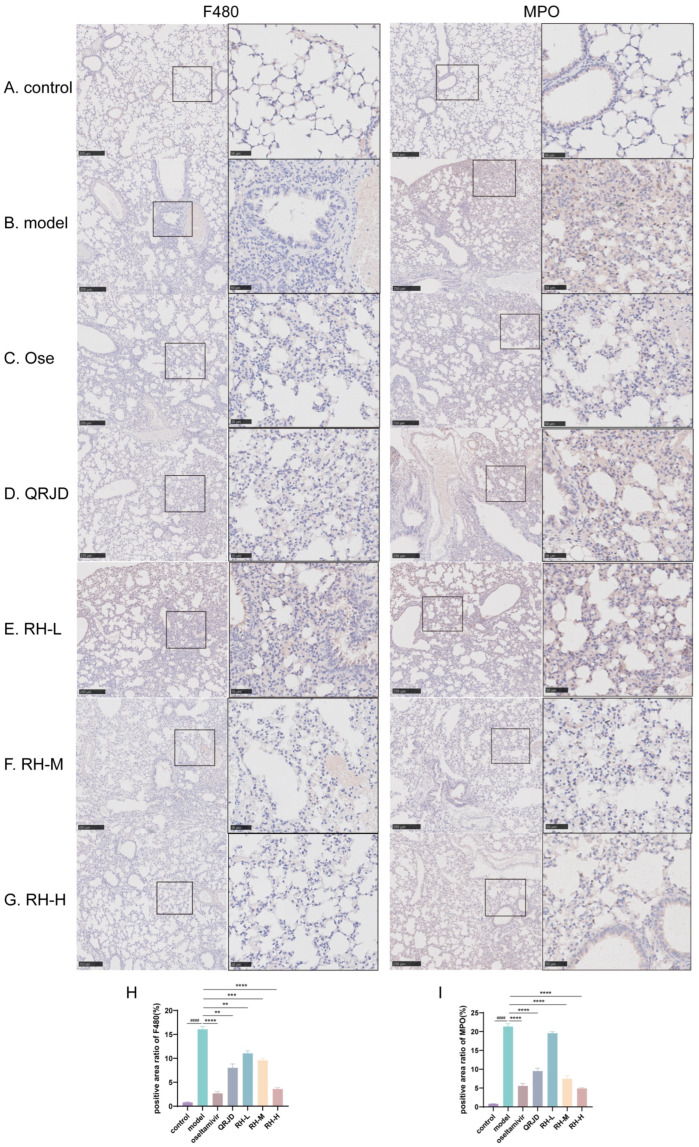
Effect of RHDS on the expression levels of F4/80, MPO in mice infected with H1N1 (n = 3) (Scale bar: 250 μm, 50 μm): (**A**) control group; (**B**) model group; (**C**) oseltamivir group; (**D**) QRJD oral solution group; (**E**) RH-L group; (**F**) RH-M group; (**G**) RH-H group; (**H**) quantitative analysis of F4/80 positive cells; (**I**) quantitative analysis of MPO positive cells. The above data are expressed as means ± SD; ^####^ *p* < 0.0001 vs. control group; ** *p* < 0.01, *** *p* < 0.001, and **** *p* < 0.0001 vs. model group.

To further examine the therapeutic effect of RHDS on H1N1 viral pneumonia, immunohistochemistry was employed to detect the protein expression levels of MPO and F4/80 in lung tissues. The tissue localisation of MPO, a neutrophil-specific granzyme, can quantitatively reflect the degree of neutrophil infiltration [32]. In the process of inflammation, activated neutrophils release MPO through a process known as degranulation. This results in the production of hypochlorous acid (HOCl), which plays a crucial role in eliminating pathogens. Additionally, the release of pro-inflammatory mediators, such as IL-6 and TNF-α, accompanies this process [33]. Quantitative analysis demonstrated that the percentage of MPO-positive areas was significantly increased in the model group compared to the control group (*p* < 0.0001). In contrast, the rate of MPO-positive regions was reduced considerably after RHDS administration (*p* < 0.0001 vs. model group). F4/80 is a mouse macrophage-specific surface glycoprotein responsible for the recognition of macrophages and the distinction between different subpopulations of macrophages [34]. The application of immunohistochemistry in detecting F4/80 provides a means to visualize the distribution and quantity of macrophages within tissues, thereby facilitating the determination of the site and extent of inflammation. Quantitative analysis demonstrated that the percentage of F4/80-positive areas in the model group was significantly increased compared to the control group (*p* < 0.0001) (Figure 6H). In addition, F4/80 protein expression was significantly reduced in the RH-L, M, and H groups compared to the model group (*p* < 0.01 or *p* < 0.0001) (Figure 6). It is suggested that the administration of RHDS significantly reduced F4/80 and MPO protein expression levels in mouse lung tissues and inhibited neutrophil over-recruitment and macrophage infiltration.

### 3.6. RHDS Treats H1N1-Infected Mice by Modulating the GPX4/SLC7A11/Nrf2 Signaling Pathway

**Figure 7:**
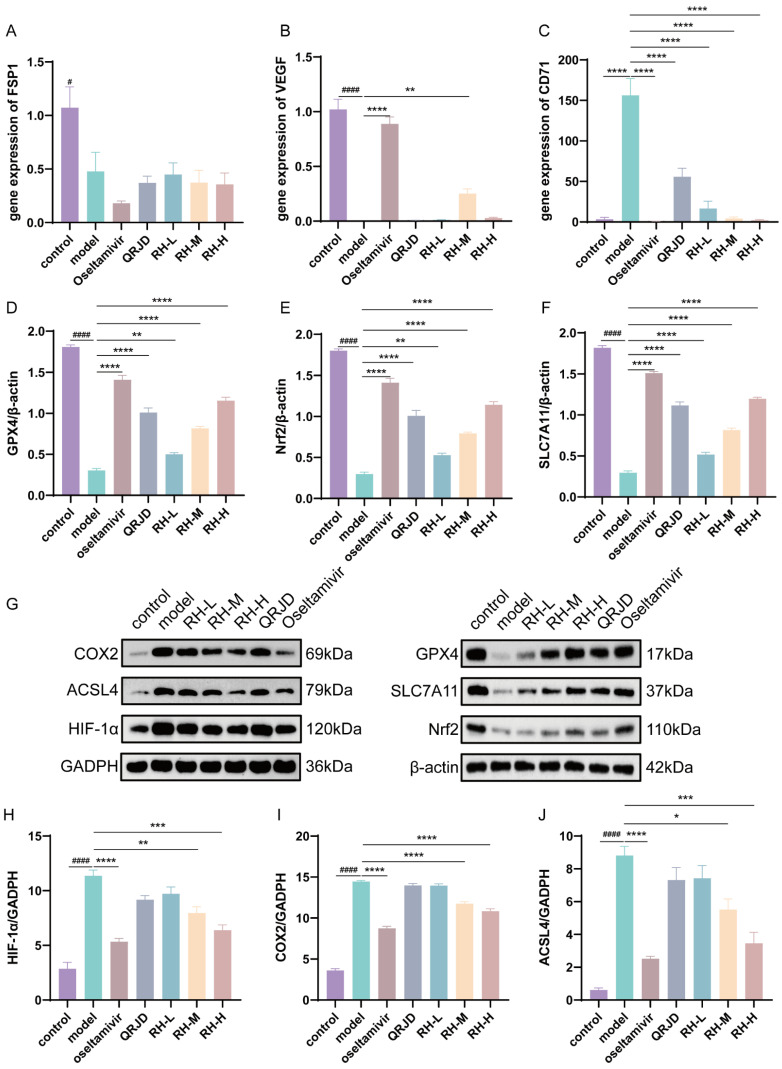
Effect of RHDS on the expression levels of key genes and proteins on the GPX4/SLC7A11/Nrf2 pathway in mice infected with H1N1: (**A**) the level of FSP1 gene mRNA; (**B**) the level of VEGF gene mRNA; (**C**) the level of CD71 gene mRNA; (**D**–**F**) the relative expression level of GPX4, Nrf2, and SLC7A11 (*n* = 3); (**G**) Images of the expression of proteins; (**H**–**J**) the relative expression level of HIF-1α, COX2, and ACSL4 (*n* = 3). The above data are expressed as means ± SD; ^#^ *p* < 0.05 and ^####^ *p* < 0.0001 vs. control group; * *p* < 0.05, ** *p* < 0.01, *** *p* < 0.001, and **** *p* < 0.0001 vs. model group.

Network pharmacology analysis was employed to assess the regulatory effects of RHDS on the HIF-1α pathway and the key pathway of ferroptosis, GPX4/SLC7A11/Nrf2, in H1N1-infected mice. The present study employed RT-qPCR in combination with Western blot experiments for the evaluation process. Western blot assay was conducted on the key proteins associated with iron-dependent cell death, including GPX4/SLC7A11/Nrf2, as well as HIF-1α protein expression (Figure 7G). The results demonstrated that H1N1 infection significantly inhibited the expression of the proteins that are negatively regulated by iron-dependent cell death, including GPX4, SLC7A11, and Nrf2, while concomitantly up-regulating HIF-1α, COX2, and ACSL4 (Figure 7D–J). Following the administration of RHDS, there was an observed up-regulation in the protein expression levels of GPX4, SLC7A11, and Nrf2, while HIF-1α, COX2, and ACSL4 protein expression levels were suppressed in a dose-dependent manner. To detect the mRNA expression levels of FSP1, VEGF, and CD71, qPCR was performed (Figure 7A,B). qPCR significantly increased the mRNA expression of VEGF in RH-M and significantly decreased the mRNA expression of the iron metabolism marker, CD71, in RH-L, M, and H, as compared with the model group. It has been proposed that the efficacy of RHDS in protecting lung tissue is attributable to its ability to modulate angiogenesis and ferroptosis inhibition. In summary, the hypothesis posits that RHDS reduces the expression of ACSL4 through the activation of Nrf2, increasing the expression of GPX4 and SLC7A11. This process leads to the scavenging of lipid ROS, thereby reducing ACSL4 expression. In addition to mitigating the hypoxic microenvironment and inflammatory factor storm by down-regulating HIF-1α and COX2 to attenuate the inflammatory injury triggered by H1N1, the following mechanisms are in place to inhibit iron accumulation: CD71 expression is reduced, and the iron accumulation is reduced through a pathway independent of HIF by up-regulating VEGF to promote tissue repair.

## 4. Discussion

IAV has emerged as a significant public health concern, posing a persistent challenge to clinical infection prevention and control due to its remarkable transmission efficiency and multidrug resistance. The pathological damage caused by IAV infection encompasses both direct cellular lesions induced by the virus and an excessive inflammatory response and cell death mechanism induced by the infection. Research has demonstrated that the influenza virus instigates innate and adaptive immune responses by infecting epithelial cells, endothelial cells, and alveolar macrophages. The overproduction of pro-inflammatory factors and a disruption in anti-inflammatory regulatory mechanisms can precipitate a cytokine storm. Consequently, this may induce apoptosis in alveolar epithelial cells, leading to lung tissue injury. Therefore, this sequence of events can lead to immunopathological damage and an elevated risk of recurrence [35]. Ferroptosis is a regulated cell death (RCD) whose central mechanism involves inhibiting GPX4 activity and accumulating lipid peroxidation products [36]. IAV infection has been demonstrated to induce iron-dependent death of alveolar epithelial cells, exacerbate inflammatory lung injury, and enhance viral replication [13,14]. Furthermore, the accumulation of ROS, a metabolite of ferroptosis, has been shown to activate NLRP3 inflammatory vesicles further and promote the release of pro-inflammatory factors. It has been demonstrated that this process instigates an array of inflammatory responses, thereby intensifying the detrimental effects of influenza virus-induced lung injury [37,38].

In this study, we systematically elucidated the potential mechanism of action of the Chinese herbal formula RHDS against H1N1 by integrating network pharmacology and ex vivo experiments. Multi-database mining and molecular docking were used to identify 32 core therapeutic targets intersecting with IAV infection, the ferroptosis pathway, and RHDS bioactive components. The present study demonstrated functional enrichment in HIF-1α signaling and STAT3-mediated immunomodulatory pathways. Research has shown a role for HIF-1α in the regulation of pro-inflammatory factors during the inflammatory response and promotes viral replication in H1N1 virus infection [39,40]. Quercetin, lignans, and kaempferol were identified as the primary bioactive constituents of RHDS, and preliminary studies have demonstrated their potential pharmaceutical activities, including anti-inflammatory, antioxidant, and antiviral properties. In the initial phases of influenza infection, quercetin demonstrated an inhibitory effect, modulating the expression of pivotal viral proteins to curtail IAV replication [41]. Recent studies have demonstrated the potential of flavonoids, such as lignans and kaempferol, in suppressing viral NA proteins and mitigating IAV-induced lung injury [42,43]. The GPX4/SLC7A11/Nrf2 pathway represents a pivotal molecular nexus in regulating ferroptosis. Dysfunction in this pathway is associated with viral infections, inflammatory injury, and organ failure. GPX4 regulates ferroptosis and controls glutathione (GSH) to maintain redox. A reduction or inactivation of GPX4 can result in iron apoptosis [44]. SLC7A11 functions as a critical component in the intracellular biosynthesis of GSH, providing essential cofactors for GPX4 (glutathione-dependent antioxidant) through the facilitation of cystine uptake and glutamate efflux [45]. Nrf2 is pivotal in regulating cellular antioxidant responses and maintaining intracellular iron homeostasis through its regulation of iron metabolism-related genes. The activation of Nrf2 can either directly or indirectly regulate the expression of GPX4 [46]. The present studies concentrate on key targets such as GPX4, SLC7A11, and Nrf2.

Multiple traditional Chinese medicines have been clinically and experimentally proven to have therapeutic effects on influenza [47,48]. RHDS has been shown to demonstrate multi-target synergistic effects when compared to commonly used antiviral TCM preparations, including Lianhua Qingwen, Huangliang Xiangru Decoction, and Yin-Qiao-San. Research on Lianhua Qingwen suggests that it may contribute to a reduction in lung damage by inhibiting viral adsorption and down-regulating the NF-κB inflammatory pathway [49]. HOODT exerts antiviral and anti-inflammatory effects by regulating the JAK/STAT signaling pathway [50]. The HXD treatment of H1N1 viral pneumonia involves the enhancement of immune function and the down-regulation of the TLR7 signaling pathway [51]. The experimental results of this study suggest that RHDS exerts synergistic antiviral effects through anti-inflammatory mechanisms, inhibition of viral replication, and regulation of the ferroptosis pathway. In vivo findings in mice demonstrated that H1N1 infection induced ferroptosis in alveolar epithelial cells by down-regulating the expression of protein levels of Nrf2 and SLC7A11 in lung tissues and inhibiting GPX4 activity. Simultaneously, viral infection activated the COX2 and ACSL4 pathways, which promoted the release of inflammatory mediators and suggested the onset of ferroptosis. The up-regulation of HIF-1α protein expression, in synergy with COX2 and ACSL4, has been demonstrated to exacerbate virus-induced inflammation and oxidative stress, while promoting viral replication. Following the administration of RHDS, activating the Nrf2/SLC7A11/GPX4 axis hindered this process, while concomitantly inhibiting ACSL4-mediated reprogramming of lipid metabolism and HIF-1α-dependent iron overload. The Nrf2/SLC7A11/GPX4 axis has been demonstrated to impede COX2 inflammatory signaling, thereby attenuating inflammatory infiltration in mouse lung tissue. This attenuation led to a reduction in viral load and lung index in lung tissue, and ultimately, ameliorated viral pneumonia. Furthermore, in vitro findings indicate that RHDS exhibits stage-dependent antiviral efficacy, with high viral suppression being achieved with therapeutic administration. In this study, we conducted a preliminary analysis and prediction of the network pharmacology of RHDS. Further investigations in vivo and in vitro have indicated that RHDS exerts an anti-H1N1 pharmacodynamic effect, with the specific mechanism being associated with the modulation of the GPX4/SLC7A11/Nrf2 pathway. However, despite the promising efficacy observed in mice, translating these findings to human clinical application faces significant challenges. Direct evidence for the efficacy and safety of RHDS in humans is currently lacking, necessitating rigorous evaluation in human-relevant models and Phase I clinical trials to assess its true translational potential. Furthermore, while the data implicate ferroptosis modulation via the Nrf2/SLC7A11/GPX4 axis pathways in the protective effect of RHDS, more direct mechanistic validation is required. Future studies should employ specific ferroptosis inhibitors or utilize GPX4 knockout mice to conclusively demonstrate the indispensable role of ferroptosis inhibition in mediating the anti-H1N1 and anti-pneumonia effects of RHDS.

## Figures and Tables

**Table 1:** Sequences of Primers Used for qPCR.

Gene Name	Forward (5′–3′)	Reverse (5′–3′)
NP	GTCAGAATGATCAAACGTGGGA	TACGGCAGGTCCATACACACAG
FSP1	GTTCAAGATGGGGTCCCAGG	CACCAGCATGAAGGGGACAT
VEGF	ACTGGACCCTGGCTTTACTG	GATCCGCATGATCTGCATGG
CD71	TGAGTGGCTACCTGGGCTAT	TTCTGGCTCAGCTGCTTGAT
GAPDH	TGGCCTTCCGTGTTCCTAC	GAGTTGCTGTTGAAGTCGCA

## Data Availability

The authors confirm that the data supporting the findings of this study are available within the article and are also available on request.
